# Dual role of injectable curcumin-loaded microgels for efficient repair of osteoarthritic cartilage injury

**DOI:** 10.3389/fbioe.2022.994816

**Published:** 2022-09-13

**Authors:** Qicai Sun, Wei Yin, Xuanliang Ru, Chun Liu, Baishan Song, Zhigang Qian

**Affiliations:** ^1^ Department of Orthopaedic Surgery, Zhejiang Hospital, Zhejiang University School of Medicine, Hangzhou, Zhejiang, China; ^2^ Zhejiang University School of Medicine, Hangzhou, Zhejiang, China

**Keywords:** Osteoarthritis, PEG-GelMA hydrogel microgel (PGMs), Chondrogenic differentiation, Inflammatory response, Cartilage regeneration

## Abstract

Curcumin has been widely used for the treatment of age-associated diseases, and showed chondroprotective potential for post-traumatic osteoarthritis (OA). However, due to the irregular-shaped and large-sized defects on joint cartilage in degenerated OA, the *in vivo* delivery and therapeutic effect of curcumin for effective repair remain challenging. In this study, we first present a PEG-GelMA [Poly(Ethylene Glycol) Dimethacrylate-Gelatin Methacrylate, PGMs] hydrogel microgel-based curcumin delivery system for both improved anti-inflammatory and pro-regenerative effects in treatment for cartilage defects. The curcumin-loaded PGMs were produced by a microfluidic system based on light-induced gelation of gelatin methacrylate (GelMA). This PGMs embedding curcumin at a relative low dosage were demonstrated to promote the proliferation and chondrogenic differentiation of mesenchymal stem cells *in vitro*. More importantly, the PGMs were shown to attenuate the inflammatory response of chondrocytes under IL-1β stimulation. Lastly, the *in vivo* application of the injectable PGMs significantly promoted the repair of large-sized cartilage injury. These results confirmed that curcumin-loaded PGMs can not only enhance the chondroprotective efficacy under inflammatory conditions but also induce efficient cartilage regeneration. This study provides an advanced strategy with anti-inflammatory and pro-regenerative dual-role therapeutic for treatment of extensive cartilage injuries.

## Introduction

Osteoarthritis (OA), which is defined by articular cartilage injuries with varying degrees, is one of the most common joint diseases worldwide (Gross et al., 2011; Chubinskaya et al., 2015). Currently, there is a lack of effective treatments for OA in clinic, and the regeneration of articular cartilage is still challenging. For patients with mild degeneration in the early stage, conservative treatments with non-steroid anti-inflammatory drugs, hyaluronic acid, and platelet-rich plasma are commonly used (Belk et al., 2021). These approaches alleviated local pathological symptoms, but cannot prevent the persistent degeneration of articular cartilage. For those irreversible degeneration and extensive destruction of articular cartilage, total joint replacement is mainly used to achieve joint structure and function restoration. However, the commonly used artificial joints based on metals and ceramics are not only with limited biological functions, but also can easily lead to infection and foreign body rejection (Veiseh and Vegas, 2019). Risks such as long-term wear and tear, and prosthesis loosening often end up with secondary surgery (Munuera Martinez, 2010).

The structural and functional reconstruction of articular cartilage based on tissue engineering technologies have shown promising future for the treatment of osteoarthritic cartilage injury. Stem cell transplantation, autologous chondrocyte transplantation, microfracture and mosaic surgery provide options for the repair of cartilage defects (Makris et al., 2015). Microfracture surgery mainly relies on bone marrow mesenchymal stem cells derived from subchondral bone to regenerate fibrocartilage-like tissue, but it is limited to small cartilage defects, and the long-term effect is not satisfactory. Autologous chondrocyte transplantation or mosaic surgery could cause additional trauma to the donor area. With a limited tissue source, the tissue integration of the transplanted cartilage and the surrounding normal tissue is poor (Kwon et al., 2019). For the patients with large cartilage defects in the moderate or late stages, there are still obvious gaps and difficulties between effective interventions and severe cartilage injuries. More importantly, in late stage OA, the chronic inflammation caused by inflammatory factors and mediators increases the extracellular matrix (ECM) degradation and impedes the further cartilage repair (Shu et al., 2020).

Curcumin, which is a natural polyphenolic compound that extracted from the turmeric, shows multiple of pharmacological activities (Alok et al., 2015). Curcumin is known for the anti-inflammation efficacy, and recently was extensively studied for the treatment of rheumatoid arthritis (Zheng et al., 2015), post-traumatic osteoarthritis (Zhang et al., 2016a), and other chronic inflammation (He et al., 2015). Therefore, these studies together suggest a promising anti-inflammatory drug for the treatment of osteoarthritic cartilage injuries. However, owing to its poor systemic bioavailability as a result of low solubility in aqueous solution, the therapeutic efficacy and translational applications of curcumin are largely impeded (Lopresti, 2018). Although nano-emulsions or nanoparticles for curcumin delivery have been developed to increase oral bioavailability, metabolic preservation (Young et al., 2014a; Dende et al., 2017), and sustained local-release (Zhang et al., 2016a), the *in vivo* pharmacokinetic and toxicology are still needed to be investigated.

Due to the irregular-shaped and large-sized defects on joint cartilage surface in late-stage of OA (Zhang et al., 2016b; Shi et al., 2017), the injury-site targeted delivery of curcumin for effective repair is still challenging. Besides, the severe degenerative microenvironments are along with a sharp decrease in endogenous reserves of stem cell numbers (Ambrosi et al., 2021) and bioactive factors (Zhang et al., 2022) for efficient cartilage repair and subchondral bone formation. While several studies suggest the implantation of scaffold materials to guide matrix synthesis or combined with growth factors to promote cartilage regeneration (Makris et al., 2015; Liu et al., 2021), high risks of infection and poor tissue integration remained to be elucidated. Novel strategies using growth factor- or cell-loaded microgel (Li et al., 2017; Lei et al., 2021) were shown as effective treatments for osteoarthritic cartilage damage through enhanced recruitment of endogenous stem cells, as well as increased nutrient, metabolite exchange, and more dynamic cell-cell and cell-material interactions (Nguyen et al., 2021). Thus, the above studies suggest that hydrogel microsphere or microgel served as a bioactive drug delivery system shows promising therapeutic approach for cartilage tissue engineering (Castro et al., 2020). Nevertheless, the treatment for OA is still challenge by the co-existence of chronic inflammation and cartilage degeneration.

In this study, we aim to determine the dual role of injectable curcumin-embedded PEG-GelMA hydrogel microgels (PGMs) on the chondroprotective efficacy under inflammatory conditions in OA progression and efficient cartilage regeneration for late-stage degeneration. Our findings showed that the curcumin-loaded PGMs at a relative low dosage was demonstrated to promote chondrogenic differentiation of mesenchymal stem cells, as well as the attenuation for inflammatory response of chondrocytes under IL-1β stimulation. In addition, the *in vivo* application of the injectable PGMs significantly enhanced the repair of large-sized cartilage injury.

## Results

### Microfluidic polymerization produces the curcumin-embedded PEG-GelMA microgels

**FIGURE 1:**
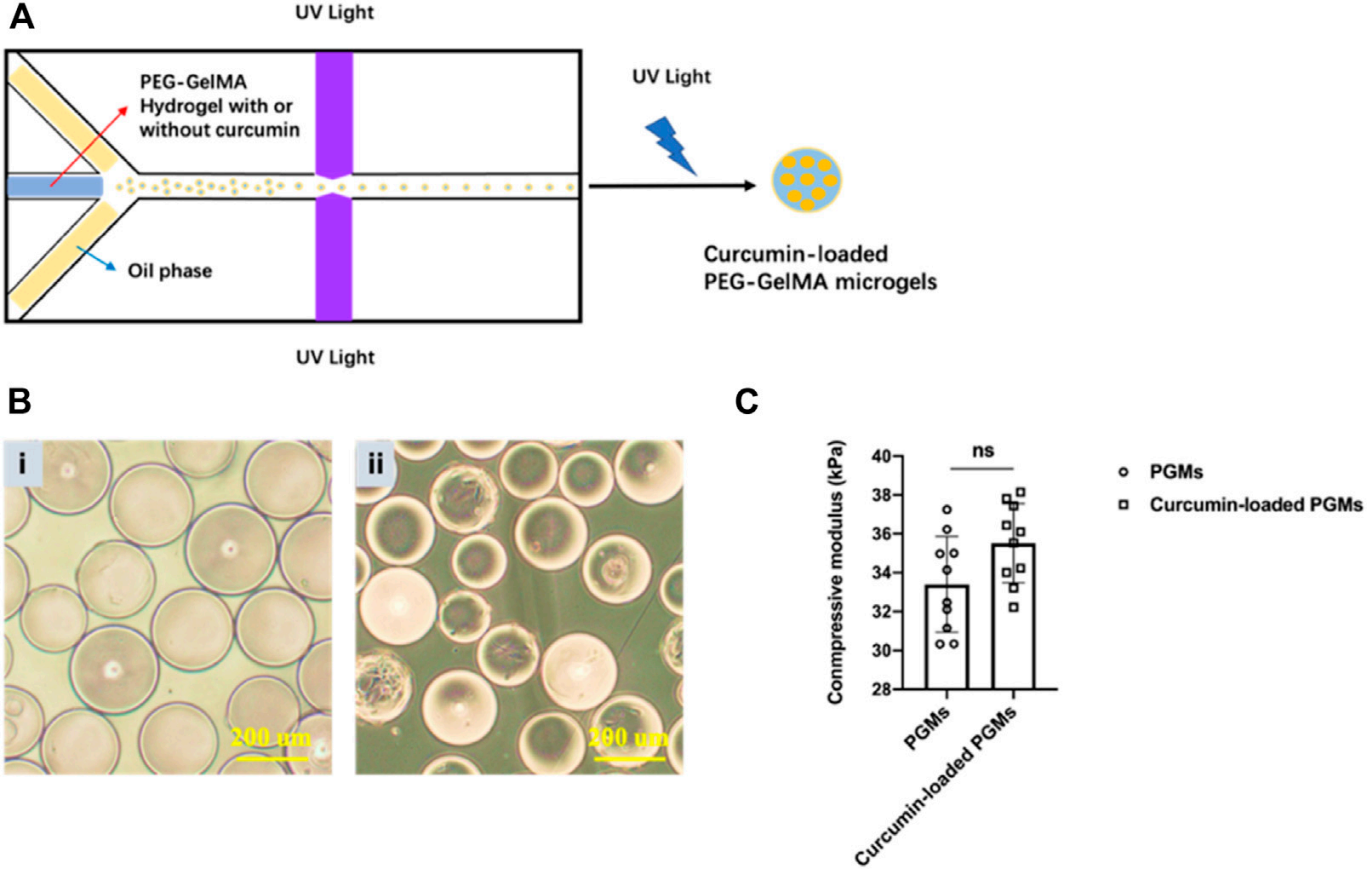
Curcumin-loaded PEG-GelMA microgel (PGMs) synthesis and characterization. **(A)** Schematic illustration of PEG-GelMA microgel fabrication by microfluidic technology. **(B)** The microscopic images at bright field of PGMs (i) and curcumin-loaded PGMs (ii), Scale bar = 200 μm. **(C)** The compressive modulus of PGMs and curcumin-loaded PGMs, respectively. *N* = 10 PGMs.

The curcumin-loaded PEG-GelMA microgels (PGMs) were fabricated by microfluidic technology and exhibited a mean diameter of 218 ± 5.25 µm (Figure 1A). From the microscopic images, we can find that the spheroids-like PGMs are highly uniform in size (Figure 1B). Previous studies have shown that the addition of GelMA to PEG increased the compressive modulus of composite hydrogels as compared to PEG alone (Hutson et al., 2011; Gao et al., 2015). In this study, the encapsulation of curcumin did not significantly affect the compressive modulus of the hydrogel microgels, as the modulus of PGMs and curcumin-loaded PGMs is about 33.396 ± 2.468 kPa and 35.513 ± 2.033 kPa, respectively (Figure 1C). These data confirmed that the compressive modulus of pure PEG hydrogels were elevated after the addition of GelMA. In addition, the PEG-GelMA microgel itself provides a comparable elastic modulus as the native articular cartilage (Beck et al., 2016).

### Curcumin-loaded PEG-GelMA hydrogel microgels enhanced the cell proliferation and chondrogenic differentiation potential of stem cells *in vitro*

**FIGURE 2:**
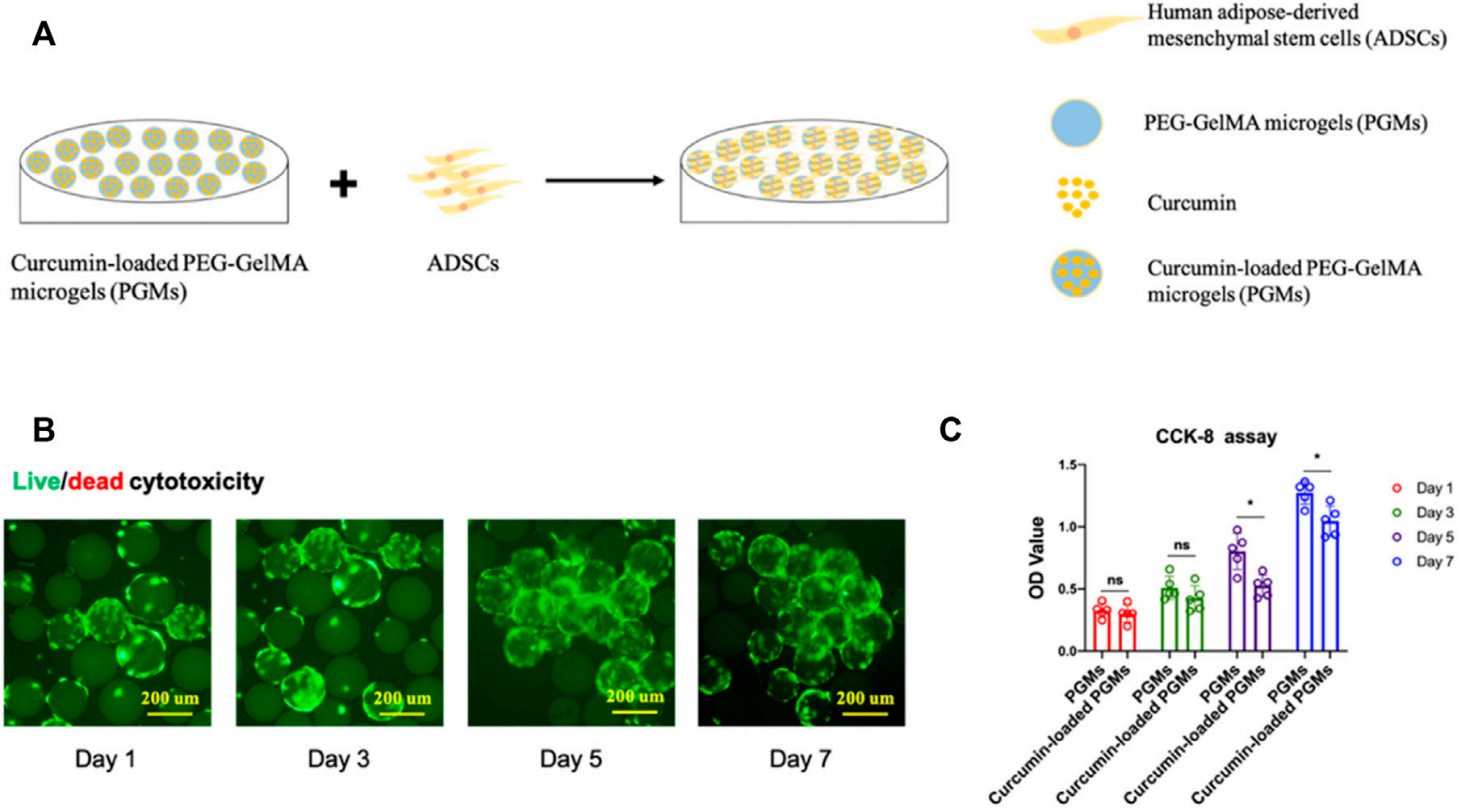
Cyto-biocompatibility and cell viability of curcumin-loaded PEG-GelMA microgel (PGMs). **(A)** Schematic of ADSCs seeding on the curcumin-loaded PEG-GelMA microgel (PGMs) for *in vitro* culture. **(B)** The Live/dead staining of ADSCs cultured on the curcumin (5 uM)-loaded PGMs, Scale bar = 200 μm. **(C)** Quantification of cell viability of ADSCs cultured on the PGMs and curcumin-loaded PGMs, respectively. Biological replicates *n* = 5.

To evaluate the cell biocompatibility of curcumin-loaded PGMs, ADSCs (Adipose-derived Mesenchymal Stem Cells) were seeded on the surface of these well-prepared PGMs for the *in vitro* culture (Figure 2A). After 1, 3, 5, and 7 days of cell expansion, live/dead staining was performed to analysis the live cell proportion across the varied time points. While dead cells were hardly detected during the culture time for 7 days, the live cells (green color-labelled) showed stable and gradually increased cell numbers (Figure 2B). To further investigate the differences on cell viability and proliferation between PGMs and the curcumin-loaded PGMs, CCK-8 assay was carried out to compare the two groups. Results of the OD values during the 7 days’ culture showed that there is no significant difference on cell proliferation at day 1 and day 3 after cell seeding, when a mild decrease of cell proliferation was found at later time points, including day 5 and day 7 (Figure 2C). These results indicate that the curcumin-loaded PGMs are available for the *in vitro* culture and normal cell proliferation, and curcumin encapsulation by PGMSs itself help maintain the cell viability.

**FIGURE 3:**
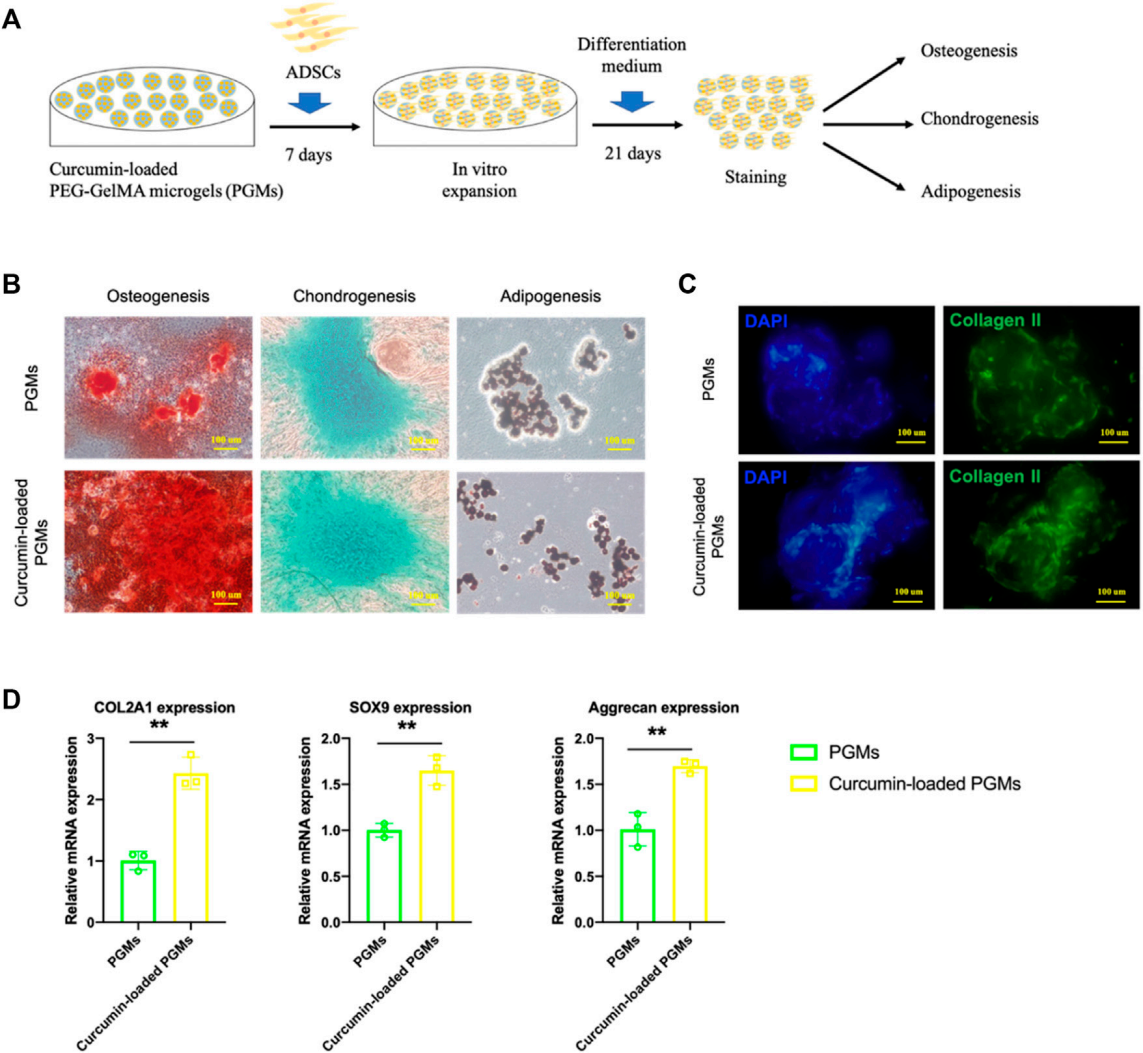
The chondrogenic bioactivity on ADSCs of curcumin-loaded PEG-GelMA microgel (PGMs). **(A)** Schematic of ADSCs culture on the curcumin-loaded PEG-GelMA microgel (PGMs) and further tri-lineage differentiation. **(B)** The level of osteogenesis, chondrogenesis, and adipogenesis of ADSCs was assessed by ARS staining, Alcian blue staining, and Oil Red staining, respectively. Scale bar = 100 μm. **(C)** Immunostaining of Collagen II protein expression in ADSCs cultured on the PGMs and curcumin (5 uM) -loaded PGMs, respectively. Scale bar = 100 μm. **(D)** Relative expression of chondrogenic marker genes (COL2A1, SOX9, Aggrecan) after chondrogenic induction for 21 days (Mean ± SD; ∗∗*p* < 0.01; biological replicates *n* = 3 per group).

We next characterized the effect of curcumin-loaded PGMs on the chondrogenic potential of mesenchymal stem cells. Human adipose-derived mesenchymal stem cells (ADSCs) were cultured either on the PGMs or curcumin-loaded PGMs system for pre-expansion for 1 week, and the differentiation capability was further tested and analyzed by tri-lineage differentiation assays (Figure 3A). After another 21 days of lineage-specific induction, various staining assays were performed for the estimation of osteogenesis, chondrogenesis, and adipogenesis, respectively. Results showed that the osteo- and chondral-lineage differentiation was improved in the curcumin-loaded PGMs group compared with the PGMs only group, while the adipogenesis didn’t show any significant difference between the two groups (Figure 3B). Besides, the immunostaining of collagen II expression, a chondrogenic marker, exhibited a much higher ratio of positive expression cells in the curcumin-loaded PGMs compared with pure PGMs (Figure 3C). Consistent with the immunostaining results, the relative mRNA expression of chondrogenic marker genes (COL2A1, SOX9, Aggrecan) was also shown greatly improved by the curcumin (Figure 3D). Collectively, the curcumin-loaded PGMs showed a good biocompatibility and cell viability, and favorable effects on promoting the chondrogenic differentiation. The above results indicated that the curcumin-loaded PGMs as bioactive microcarriers for enhanced stem cell growth hold great potential for efficient *in vivo* cartilage injury regeneration by promoting chondrogenic differentiation of joint-resident stem cells.

### Curcumin-loaded PEG-GelMA hydrogel microgels alleviate the inflammatory effects of chondrocytes

**FIGURE 4:**
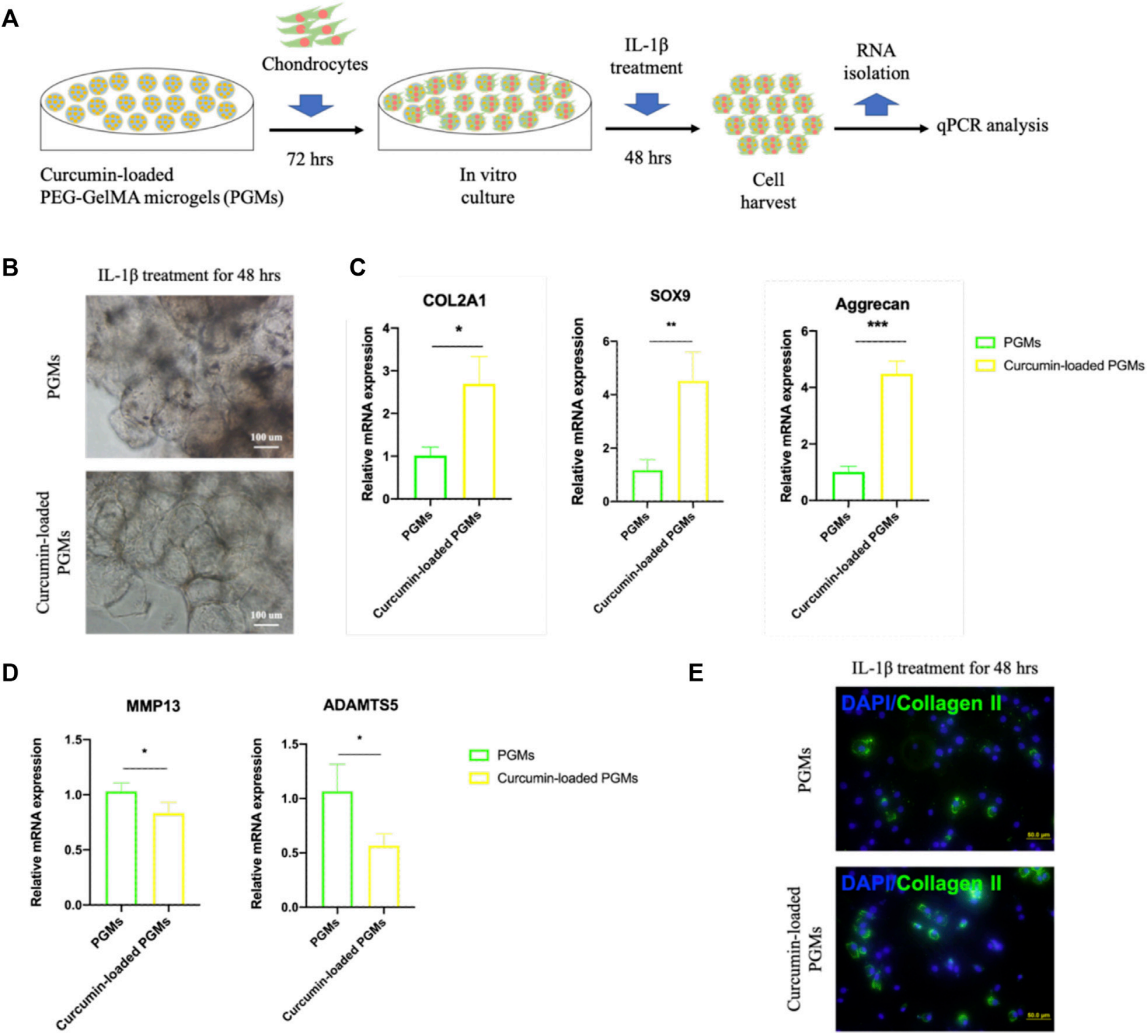
The amelioration of chondrocyte inflammation with curcumin-loaded PEG-GelMA microgel (PGMs). **(A)** Schematic of human chondrocytes cultured on the curcumin-loaded PEG-GelMA microgel (PGMs) for *in vitro* culture and further IL-1β stimulation (10 ng/ml). **(B)** The bright-field images of cultured chondrocytes after IL-1β treatment in the PGMs and curcumin (5 µM)-loaded PGMs groups, respectively. Scale bar = 100 μm. **(C)** Quantification of the expression of phenotypic genes (COL2A1, SOX9, Aggrecan) for chondrocytes, and **(D)** Quantification of the expression of inflammatory genes (MMP13, ADAMTS5) for chondrocytes. (Mean ± SD; ∗*p* < 0.05, ∗∗*p* < 0.01; ∗∗∗*p* < 0.001; biological replicates *n* = 4 per group). **(E)** Immunostaining of Collagen II protein expression of the chondrocytes (sub-cultured on cover slip for 2 days) after IL-1β treatment in the PGMs and curcumin-loaded PGMs, respectively. Scale bar = 50 μm.

To better explore the potential of curcumin-loaded PGMs against the chronic microenvironment during the osteoarthritis progression, we thus tested the anti-inflammatory effects of the curcumin-loaded PGMs on the *in vitro* cultured chondrocytes (Figure 4A). After 72 h of culture on the curcumin-loaded PGMs, the chondrocytes were subsequently treated with IL-1β for another 48 h. Interestingly, chondrocytes that cultured on the curcumin-loaded PGMs system showed apparent cell-cell contact as well as significantly fewer black spots, indicating that the loaded curcumin alleviated the cell apoptosis in respond to the IL-1β stimulation (Figure 4B).

The gene expression levels of chondrocyte phenotypic markers and inflammatory factors of chondrocytes were then detected by qRT-PCR assays. The results showed that the relative expression level of chondrocyte marker genes COL2A1, SOX9, and Aggrecan was significantly maintained in the curcumin-loaded PGMs group (Figure 4C), while the inflammation-related genes MMP13 and ADAMTS5 were significantly decreased (Figure 4D). At protein level, the Collagen II expression of chondrocytes was also found greatly increased after IL-1β stimulation in the curcumin-loaded PGMs culture system (Figure 4E). Taken together, the injectable curcumin-loaded PGMs system could not only maintain chondrocyte phenotype, but also ameliorate the local inflammatory state and matrix degeneration of the injured cartilage.

### *In vivo* application of Curcumin-loaded PEG-GelMA hydrogel microgels promoted the regeneration of large-sized cartilage defects

**FIGURE 5:**
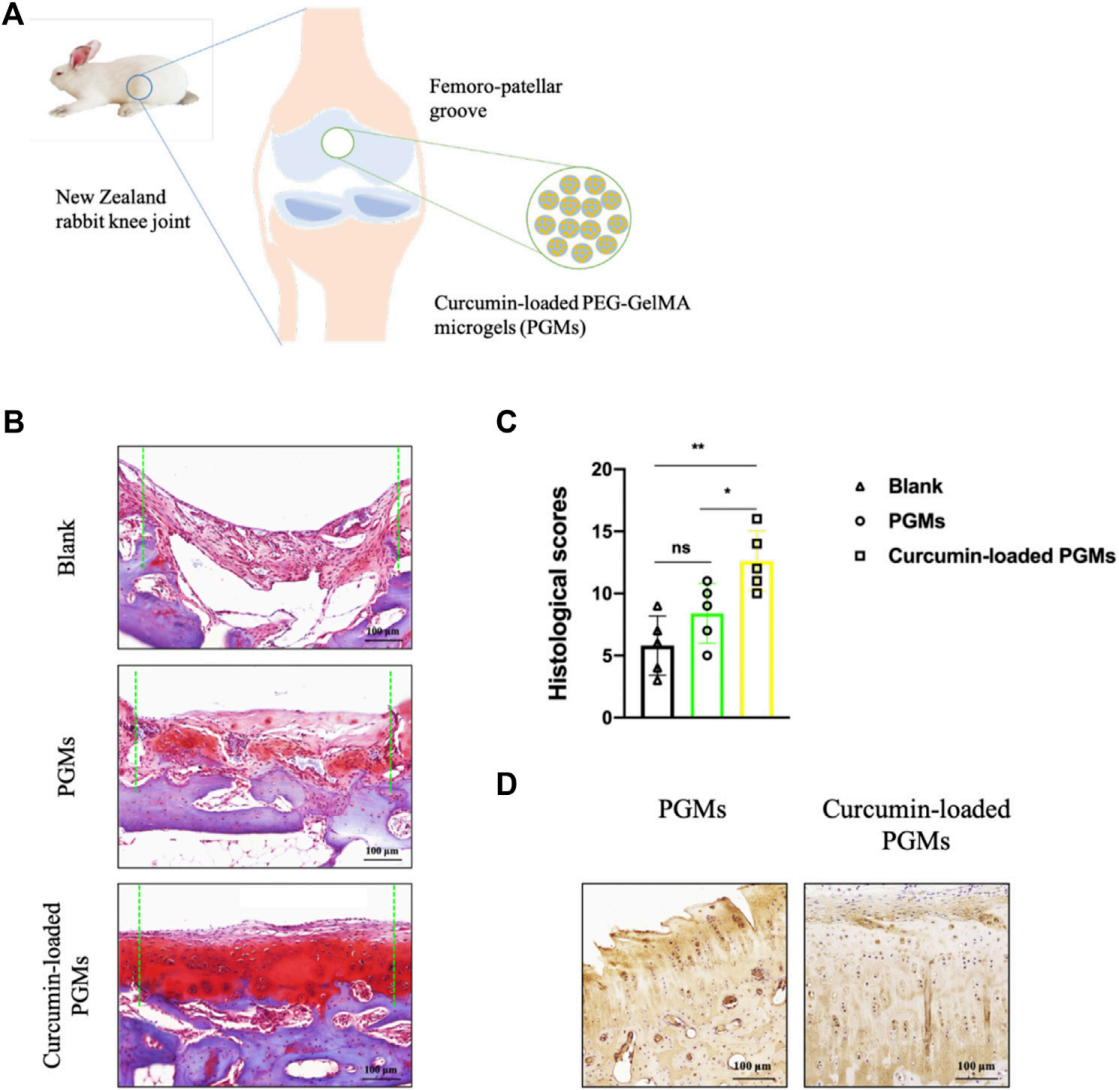
The amelioration of chondrocyte inflammation with curcumin-loaded PEG-GelMA microgel (PGMs). **(A)** Schematic of the implantation of curcumin-loaded PGMs for the repair of cartilage defects in a rabbit femoro-patellar groove. **(B)** The Safranin-O staining of the repaired cartilage tissues in Blank, PGMs and curcumin-loaded PGMs groups at 8 weeks post-surgery. Scale bar = 100 μm. **(C)** Quantification of the histological scores of cartilages under evaluation among Blank, PGMs and curcumin-loaded PGMs groups in Safranin-O staining results. (Mean ± SD; ∗*p* < 0.05, ∗∗*p* < 0.01; *n* = 5 per group). **(D)** The immunohistochemical staining of Collagen II in the repaired cartilage between PGMs and curcumin-loaded PGMs groups at 8 weeks post-surgery. Scale bar = 100 μm.

To investigate the dual role of chondroprotective efficacy under inflammatory conditions and promoting efficient cartilage regeneration when utilizing the curcumin-loaded PGMs, we performed a cartilage defect animal experiment for *in vivo* assessment. A circular defect with a 5 mm diameter was created at the femoro-patellar grooves in rabbits, and the cell-free curcumin-loaded PGMs were implanted into the defect area, while pure PGMs were also transplanted as vehicle control (Figure 5A).

Eight weeks after the surgery, the tissue samples from the defect regions were collected and analyzed. Safranin-O staining results of the tissue sections among different groups showed that the curcumin-loaded PGMs significantly improved the regeneration of injured cartilage compared with both the vehicle PGMs and the blank defect (Figure 5B). Further histological assessment of chondrocyte apoptosis and cartilage erosion that resulted from chronic inflammation showed a relatively lower level in response to the treatment of the curcumin-loaded PGMs, when compared to other groups (Figure 5C). Further immunohistochemical staining analysis on the expression of Collagen II, which is the hall-marker of chondrogenic differentiation, showed that the cartilage regeneration was enhanced by the curcumin-loaded PGMs (Figure 5D). The above results indicate that the injectable curcumin-loaded PGMs can effectively promote the cartilage regeneration either by the increased chondrogenic differentiation or the decreased level of local inflammation during the pathological degeneration.

## Discussion

Osteoarthritis (OA) is featured for co-existence of chronic inflammation and cartilage degeneration, and current treatments targeting either aspect are difficult for covering the complex conditions at the same time (Lieberthal et al., 2015). In the middle and late stages of OA progression, neither anti-inflammatory drugs nor small molecules that targeting specific molecular target alone cannot fully alleviate the pathological symptoms and prevent the progressive degeneration (An et al., 2021). This study illustrates a therapeutic strategy of the injectable curcumin-loaded PGMs with a dual role of promoting the chondroprotective efficacy under inflammatory conditions and inducing endogenous cartilage regeneration simultaneously.

The chronic inflammation during the OA development is still one of the most difficulties for the clinical treatment. Current pharmacological treatments provide symptomatic relief to joint pain and local inflammation, however, there is not a clear clinical effect on OA disease prevention or therapy. While most of the current efforts have been focused on developing novel molecular targets as well as their disease-modifying drugs, the side-effects exist regarding the non-tissue-specific and long-term use of these treatments (Yang et al., 2021). Curcumin has been shown to mitigate the inflammatory process by decreasing the synthesis of inflammatory mediators such as interleukin (IL)-1β, tumor necrosis factor (TNF)-α, IL-6, IL-8, prostaglandin E2 (PGE2), and cyclooxygenase-2 (COX-2) (Zhang et al., 2016a; Sun et al., 2017; Sundar Dhilip Kumar et al., 2018). Moreover, curcumin suppresses the gene expression of matrix metalloproteinases (MMPs) (Zhang et al., 2016a; Mogharrabi et al., 2020) and nuclear factor kappa B (NF-kB) activation (Buhrmann et al., 2021), which play critical roles in the breakdown of the cartilage ECM. Due to the extremely limited oral bioavailability of curcumin (Ma et al., 2019), local application could provide effective delivery and absorption in treatment. More specifically, the bioavailability of curcumin at the disease site for OA treatment could be greatly enhanced by using injectable drug-delivery systems (Tiwari et al., 2017).

Nano-emulsion and nanoparticles were often taken as the promising approach for controlled release of curcumin in multiple situations (Young et al., 2014b; Zheng et al., 2015; Zhang et al., 2016a; Shi et al., 2016; Dende et al., 2017). However, the therapeutic effects are often impeded by the random distribution and extensive diffusion of drugs across the knee joint tissues. Surprisingly, the long-term toxicity profile of local distribution (Saifi et al., 2018) as well as the body wide non-targeting infiltration (Mohammadpour et al., 2019; Wu et al., 2019) of nanoparticles are still one of the most important concerns when drug-loaded nano-emusions or nanoparticles were intraarticularly injected. On the contrary, hydrogels spheroids at micro-scale were proven to be more available for the direct release of cells, growth factors and drugs (Annamalai et al., 2019; Volpatti et al., 2021), and could provide as a dense barrier for inflammatory erosion and chondrocyte apoptosis (Wei et al., 2021; Han et al., 2022).

Actually, hydrophilic microgels have been used as microcarriers for *in vitro* cell culture and *in vivo* stem cell delivery system, both the long-term cell survival and microtissue formation were shown promising when applied *in vivo* (Luo et al., 2019). Furthermore, the delayed degradation of microgels and their enough surface for further resident adipose-derived stem cell proliferation are more important for the cartilage regeneration regarding the flexible intra-articular microenvironment. In our study, the curcumin-loaded PGMs not only filled up the irregular cartilage injury, but also enhanced the joint lubrication and provided enough surface for efficient endogenous stem cell migration and adhesion. These results together suggested that the curcumin-loaded PGMs system in this study better promotes the new cartilage formation when reduces the inflammatory effects by local delivery of curcumin at a low dosage and at the same time.

The injectability of the curcumin-loaded PGMs system in this study is another advantage for promising minimal-invasive application in OA treatment. Provided with the availability for application in response to the irregular-shaped and large-sized defects on joint cartilage surface in inflammatory OA, the *in vivo* delivery of anti-inflammatory drugs by PGMs is an alternative strategy for late-staged OA treatment. Even though, further investigations are still needed to clarify the main stem cell resources and subtypes, as well as their detailed contribution for the regeneration process of inflammatory cartilage injuries.

## Conclusion

In summary, the curcumin-loaded PGMs at a relative low dosage was demonstrated to promote the proliferation and chondrogenic differentiation of mesenchymal stem cells *in vitro*. More importantly, the curcumin-loaded PGMs was shown to attenuate the inflammatory response of chondrocytes under IL-1β stimulation. Lastly, the *in vivo* application of the injectable PGMs significantly enhanced the repair of large-sized cartilage injury. These results suggest that the curcumin-loaded PGMs play a dual role in OA treatment by promoting the chondroprotective efficacy under inflammatory conditions and inducing efficient cartilage regeneration.

## Materials and methods

### Preparation of curcumin-loaded PEG-GelMA hydrogel microgels

The poly(ethylene glycol) dimethacrylate (PEGDMA, PEG)-gelatin methacrylate (GelMA) microgels were synthesized using microfluidic device as described in previous studies with minor modifications (Hutson et al., 2011; Gao et al., 2015). Briefly, curcumin powder (Sigma-Aldrich) was dissolved in 10 ml PEGDMA by stirring with a magnetic bar and heating in a boiling water bath to 100°C for 15 min until completely dissolved. After cooling, the curcumin-contained PEGDMA and GelMA were mixed together in PBS at 10% (w/v) and 5% (w/v), respectively (curcumin at a final concentration of 5 µM), in which PEGDMA did not contain curcumin was treated as vehicle control in the following experimental analysis. The aqueous phase was composed of PEG-GelMA hydrogel and 30 mg/ml photo-initiator 2-hydroxy-1 (4-(hydroxyethox)pheny)-2-methyl-1-propanone, and the continuous oil phase contained mineral oil. After starting the pumps, the aqueous and continuous phases were slowly injected into the syringes and the flow rate of the liquid in the channel is adjusted through injection pumps. Microgels were synthesized using PDMS flow-focusing devices and washed 3 times with a 1% BSA solution in PBS. The prepared hydrogel droplets were further solidified by photocrosslinking for 30 s under UV irradiation at the wavelength of 365 nm (6.9 mW/cm^2^) wavelength. Lastly, the oil and active agent on the surface of PEG-GelMA microgels were removed by repeated washing with acetone and 75% ethanol, and purified by washing with PBS for 24 h. Size distribution of the microgels was determined *via* microscopy image analysis using ImageJ.

### Mechanical testing

Samples were detached from the culture dish and incubated in PBS at room temperature. The mechanical test was performed with nano-indentation (Optics) according to the manufacturer’s protocol. The compressive modulus was determined as the slope of the linear region corresponding with 0%–10% strain.

### Cell culture

Human adipose-derived mesenchymal stem cells (ADSCs) were obtained after digesting adipose tissues acquired from donors (68 years old) with written informed consent. In brief, fat pad pieces (1–2 mm^3^) were digested with collagenase (Sigma-Aldrich) at 37°C for 2 h. The isolated cells were cultured in L-DMEM (Gibco) with 10% fetal bovine serum and 1% penicillin/streptomycin (Life Technologies) at 37°C and 5% CO2. The medium was changed every 3 days, and cells at passage 5 were used in this study.

Primary chondrocytes were obtained from patients undergoing total joint replacement surgery (aged 68–81 years) were cultured in F12 with 10% fetal bovine serum and 1% penicillin/streptomycin. F12 with 1% fetal bovine serum overnight were used for starvation prior to curcumin treatment. Chondrocytes were incubated with IL-1β (10 ng/ml, Sigma) for 48 h after incubation with curcumin-contained medium for 72 h (Zhang et al., 2016a). Cells were then lysed and RNA isolated for further analysis.

### Cell live/dead staining

ADSCs that co-cultured with PEG-GelMA hydrogel microgels (PGMs) was tested by live/dead cytotoxicity kit. The ADSCs were co-cultured with PGMs at a density of 4 × 10^5^ cells/mL. Then, the ADSC/PGMs suspension was mixed and seeded in a 24-well plate, and cultured in humidified incubator containing 5% CO_2_ at 37°C. After culturing for 1, 3, 5, and 7 days, cells were stained with the live/dead cytotoxicity kit and imaged with a confocal laser scanning microscope (OLYMPUS IX83-FV1000).

### Cell viability

ADSCs between passages 3–5 were cultured with PGMs at a density of 1 × 10^4^ cells/disc in a 96-well plate. 33 µl medium containing microgel was added to each well, and then added complete medium to 200 µl. The proliferation of ADSCs were evaluated by the Cell Counting Kit-8 (CCK-8) testing. At 1, 3, 5, and 7 days, the medium was replaced with 180 μl of fresh complete medium and 20 μl of CCK-8 reagent (Dojindo, Japan) each well and incubated for 2 h at 37°C with 5% CO_2_. Finally, the staining solution was collected and measured using a multi-plate reader at 450 nm.

## Quantitative real-time PCR

Quantitative real-time PCR was carried out on 5 days to evaluate the expression of chondrogenic marker genes (SOX9, MMP13, and ADAMTS5) in cells co-cultured with PGMs and curcumin-loaded PGMs. Quantitative real-time PCR was performed with SYBR^®^ Premix Ex Taq™ (Takara) using a ABI 7500 Sequencing Detection System (Applied Biosystems, Foster City, United States). GAPDH was used as a house-keeping. Data were analyzed using the comparison Ct (2^−ΔΔCt^) method and expressed as fold changes compared to the control.

The sequences of the primers used are listed in Table 1.

**TABLE 1:** Primers used in the qRT-PCR assay.

Gene	Forward (5–3′)	Reverse (5–3′)
GAPDH	GGC​AAG​TTC​AAC​GGC​ACA​G	CGC​CAG​TAG​ACT​CCA​CGA​CAT
SOX9	CTG​ACC​GTG​ACC​GTA​GCA​AGT	TGG​ATG​TGG​GCT​TTG​GAC​TCA
COL2A1	GTC​TGT​GAC​ACT​GGG​ACT​GT	TCT​CCG​AAG​GGG​ATC​TCA​GG
Aggrecan	CTG​CAG​ACC​AGG​AGG​TAT​GTG​A	GTT​GGG​GCG​CCA​GTT​CTC​AAA​T
MMP13	ATG​CAG​TCT​TTC​TTC​GGC​TTA​G	ATG​CCA​TCG​TGA​AGT​CTG​GT
ADAMTS5	ATCAC-CCAATGCCAAGG	AGCAGAGTAGGAGACAAC

### Trilineage differentiation assays

For osteogenesis, the cells were cultured in osteogenic medium containing α-DMEM with 10% FBS supplemented with 0.1 μM dexamethasone, 0.2 mM L-ascorbic acid, and 10 mM glycerol 2-phosphate disodium salt hydrate. The medium was changed every 2 days. For chondrogenesis, the cells were resuspended at a concentration of 5 × 10^5^ cells in 200 μl of growth media and plated as micro-mass. After 2 h at 37°C, the micro-mass were covered with chondrogenic medium containing DMEM with 10% FBS supplemented with 0.1 μM dexamethasone, 100 μg/ml sodium pyruvate, 40 μg/ml L-proline, 50 μg/ml L-ascorbic acid, 50 mg/ml ITS, and 10 ng/ml TGFβ1. The medium was changed every 2 days for 21 days. For adipogenesis, the confluent cells were cultured with adipogenic medium containing α-DMEM with 10% FBS supplemented with 10 μg/ml insulin, 100 μM indomethacin, 0.5 mM 3-iso- butyl-1-methylxanthine, and 0.1 μM dexamethasone. The medium was changed every 2 days.

### Immunofluorescent staining

Cells cultured on PGMs system or cover slips were fixed with 4% paraformaldehyde (PFA) for 30 min and then permeabilized with 0.01% Triton X-100 for 10 min at room temperature. After washed with PBS for 3 times, cells were blocked with 1% bovine serum albumin for 1 h at room temperature. The primary antibody (Anti-Collagen II antibody, diluted 200-fold, ab34712, Abcam) was incubated at 4°C overnight. Cells were next incubated with secondary antibody (diluted 200-fold, G-Rabbit Alexa Fluor^®^ 488, A11008; Invitrogen) for 1 h at room temperature. After incubation, the nuclei were lastly stained with 1 X DAPI. After staining, the cells were observed using a confocal microscope (OLYMPUS IX83-FV1000).

### For alizarin red staining

Cells were washed with cold PBS and fixed with 4% PFA for 30 min on ice. Cells were washed with distilled water and stained with 2% alizarin red solution for 15 min. Cells were then washed thoroughly with distilled water and air dried before microscopic observation.

### Alcian blue staining

After culture medium was removed, and cells were washed with PBS and fixed with 4% PFA for 30 min. Cells were stained for 30 min with 1% Alcian Blue solution 8GX (Sigma) in 3% acetic acid, pH 2.5, and washed three times with 0.1 N HCl and then washed three times with PBS.

### Oil red staining

After culture medium was removed, and cells were washed with PBS and fixed with 4% PFA for 30 min at room temperature. Cells were rinsed again with PBS and stained for 30 min with Oil Red O working solution. Cells were then observed under a light microscope after three times of washes with PBS.

### Animal experiments

All animals were treated according to standard guidelines approved by the Zhejiang University Ethics Committee (NO. ZJU20220320). Adult New Zealand white rabbits (3 kg, Male, 15 rabbits, 10 weeks old) were used in this study. All surgeries were performed under general anesthesia. The knee joint was opened with medial para-patellar approach. The patella was dislocated laterally and the surface of the femoro-patellar groove was exposed. Cartilage defects were created using a custom-made scalpel with a thickness of 2 mm. PGMs and curcumin-loaded PGMs were applied to the surface of the cartilage defects, separately (*n* = 5 rabbits in each group). The non-treated blank (*n* = 5 rabbits) was treated as control group. 8 weeks after surgery, animals were sacrificed and the joint cartilage samples were harvested for further analysis.

### Histological analysis

Tissue samples were fixed in 4% paraformaldehyde and decalcified in 10% EDTA for 4 weeks. Paraffin sections were stained with Safranin-O staining and histological observations were performed under a light microscope. The repaired cartilage tissue was graded by an established histological scoring system, according to a modified O’Driscoll histology scoring (MODS) system (Kleemann et al., 2005).

### Statistical analysis

The data of this study were averaged ±standard deviation (SD) of statistical data using SPSS software. At least three parallel samples (*n* = 3) were set for all experiments. Comparison between the two groups was analyzed by the independent *t* test, and *p* < 0.05 was considered statistically significant. * indicates *p* < 0.05, ** indicates *p* < 0.01, ***p < 0.001.

## Data Availability

The raw data supporting the conclusion of this article will be made available by the authors, without undue reservation.

## References

[B1] Alok A. Singh I. D. Singh S. Kishore M. Jha P. C. (2015). Curcumin - pharmacological actions and its role in oral submucous fibrosis: A review. J. Clin. Diagn. Res. 9 (10), ZE01–3. 10.7860/JCDR/2015/13857.6552 PMC4625352 26557633

[B2] Ambrosi T. H. Marecic O. McArdle A. Sinha R. Gulati G. S. Tong X. M. (2021). Aged skeletal stem cells generate an inflammatory degenerative niche. Nature 597 (7875), 256–262. 10.1038/s41586-021-03795-7 34381212 PMC8721524

[B3] An J. S. Tsuji K. Onuma H. Araya N. Isono M. Hoshino T. (2021). Inhibition of fibrotic changes in infrapatellar fat pad alleviates persistent pain and articular cartilage degeneration in monoiodoacetic acid-induced rat arthritis model. Osteoarthr. Cartil. 29 (3), 380–388. 10.1016/j.joca.2020.12.014 33388431

[B4] Annamalai R. T. Hong X. Schott N. G. Tiruchinapally G. Levi B. Stegemann J. P. (2019). Injectable osteogenic microtissues containing mesenchymal stromal cells conformally fill and repair critical-size defects. Biomaterials 208, 32–44. 10.1016/j.biomaterials.2019.04.001 30991216 PMC6500486

[B5] Beck E. C. Barragan M. Tadros M. H. Gehrke S. H. Detamore M. S. (2016). Approaching the compressive modulus of articular cartilage with a decellularized cartilage-based hydrogel. Acta Biomater. 38, 94–105. 10.1016/j.actbio.2016.04.019 27090590 PMC4903909

[B6] Belk J. W. Kraeutler M. J. Houck D. A. Goodrich J. A. Dragoo J. L. McCarty E. C. (2021). Platelet-rich plasma versus hyaluronic acid for knee osteoarthritis: A systematic review and meta-analysis of randomized controlled trials. Am. J. Sports Med. 49 (1), 249–260. 10.1177/0363546520909397 32302218

[B7] Buhrmann C. Brockmueller A. Mueller A. L. Shayan P. Shakibaei M. (2021). Curcumin attenuates environment-derived osteoarthritis by Sox9/NF-kB signaling Axis. Int. J. Mol. Sci. 22 (14), 7645. 10.3390/ijms22147645 34299264 PMC8306025

[B8] Castro L. M. M. Sequeira A. Garcia A. J. Guldberg R. E. (2020). Articular cartilage- and synoviocyte-binding poly(ethylene glycol) nanocomposite microgels as intra-articular drug delivery vehicles for the treatment of osteoarthritis. ACS Biomater. Sci. Eng. 6 (9), 5084–5095. 10.1021/acsbiomaterials.0c00960 33455260 PMC8221079

[B9] Chubinskaya S. Haudenschild D. Gasser S. Stannard J. Krettek C. Borrelli J. Jr. (2015). Articular cartilage injury and potential remedies. J. Orthop. Trauma 29 (12), S47–S52. 10.1097/bot.0000000000000462 26584267 PMC7054985

[B10] Dende C. Meena J. Nagarajan P. Nagaraj V. A. Panda A. K. Padmanaban G. (2017). Nanocurcumin is superior to native curcumin in preventing degenerative changes in Experimental Cerebral Malaria. Sci. Rep. 7, 10062. 10.1038/s41598-017-10672-9 28855623 PMC5577147

[B11] Gao G. Schilling A. F. Hubbell K. Yonezawa T. Truong D. Hong Y. (2015). Improved properties of bone and cartilage tissue from 3D inkjet-bioprinted human mesenchymal stem cells by simultaneous deposition and photocrosslinking in PEG-GelMA. Biotechnol. Lett. 37 (11), 2349–2355. 10.1007/s10529-015-1921-2 26198849

[B12] Gross K. D. Niu J. B. Stefanik J. J. Guermazi A. Roemer F. Sharma L. (2011). Breaking the law of valgus: The surprising and unexplained prevalence of medial patellofemoral cartilage damage. Arthritis Rheumatism 63 (10), S634–S635. 10.1136/annrheumdis-2011-200606 PMC4011177 22534825

[B13] Han Z. Y. Bai L. Zhou J. Qian Y. H. Tang Y. K. Han Q. B. (2022). Nanofat functionalized injectable super-lubricating microfluidic microspheres for treatment of osteoarthritis. Biomaterials 285, 121545. 10.1016/j.biomaterials.2022.121545 35512418

[B14] He Y. Yue Y. Zheng X. Zhang K. Chen S. Du Z. (2015). Curcumin, inflammation, and chronic diseases: How are they linked? Molecules 20 (5), 9183–9213. 10.3390/molecules20059183 26007179 PMC6272784

[B15] Hutson C. B. Nichol J. W. Aubin H. Bae H. Yamanlar S. Al-Haque S. (2011). Synthesis and characterization of tunable poly(ethylene glycol): Gelatin methacrylate composite hydrogels. Tissue Eng. Part A 17 (13-14), 1713–1723. 10.1089/ten.tea.2010.0666 21306293 PMC3118706

[B16] Kleemann R. U. Krocker D. Cedraro A. Tuischer J. Duda G. N. (2005). Altered cartilage mechanics and histology in knee osteoarthritis: Relation to clinical assessment (ICRS grade). Osteoarthr. Cartil. 13 (11), 958–963. 10.1016/j.joca.2005.06.008 16139530

[B17] Kwon H. Brown W. E. Lee C. A. Wang D. A. Paschos N. Hu J. C. (2019). Surgical and tissue engineering strategies for articular cartilage and meniscus repair. Nat. Rev. Rheumatol. 15 (9), 550–570. 10.1038/s41584-019-0255-1 31296933 PMC7192556

[B18] Lei Y. T. Wang Y. P. Shen J. L. Cai Z. W. Zeng Y. S. Zhao P. (2021). Stem cell-recruiting injectable microgels for repairing osteoarthritis. Adv. Funct. Mat. 31 (48), 2105084. 10.1002/adfm.202105084

[B19] Li F. Y. Truong V. X. Thissen H. Frith J. E. Forsythe J. S. (2017). Microfluidic encapsulation of human mesenchymal stem cells for articular cartilage tissue regeneration. ACS Appl. Mat. Interfaces 9 (10), 8589–8601. 10.1021/acsami.7b00728 28225583

[B20] Lieberthal J. Sambamurthy N. Scanzello C. R. (2015). Inflammation in joint injury and post-traumatic osteoarthritis. Osteoarthr. Cartil. 23 (11), 1825–1834. 10.1016/j.joca.2015.08.015 PMC4630675 26521728

[B21] Liu Y. Z. Peng L. Q. Li L. L. Huang C. S. Shi K. D. Meng X. B. (2021). 3D-bioprinted BMSC-laden biomimetic multiphasic scaffolds for efficient repair of osteochondral defects in an osteoarthritic rat model. Biomaterials, 279, 121216. 10.1016/j.biomaterials.2021.121216 34739982

[B22] Lopresti A. L. (2018). The problem of curcumin and its bioavailability: Could its gastrointestinal influence contribute to its overall health-enhancing effects? Adv. Nutr. 9 (1), 41–50. 10.1093/advances/nmx011 29438458 PMC6333932

[B23] Luo C. Fang H. Zhou M. Li J. Zhang X. Liu S. (2019). Biomimetic open porous structured core-shell microtissue with enhanced mechanical properties for bottom-up bone tissue engineering. Theranostics 9 (16), 4663–4677. 10.7150/thno.34464 31367248 PMC6643438

[B24] Ma Z. Wang N. He H. Tang X. (2019). Pharmaceutical strategies of improving oral systemic bioavailability of curcumin for clinical application. J. Control. Release 316, 359–380. 10.1016/j.jconrel.2019.10.053 31682912

[B25] Makris E. A. Gomoll A. H. Malizos K. N. Hu J. C. Athanasiou K. A. (2015). Repair and tissue engineering techniques for articular cartilage. Nat. Rev. Rheumatol. 11 (1), 21–34. 10.1038/nrrheum.2014.157 25247412 PMC4629810

[B26] Mogharrabi M. Rahimi H. R. Hasanzadeh S. Dastani M. Kazemi-Oskuee R. Akhlaghi S. (2020). The effects of nanomicelle of curcumin on the matrix metalloproteinase (MMP-2, 9) activity and expression in patients with coronary artery disease (cad): A randomized controlled clinical trial. ARYA Atheroscler. 16 (3), 136–145. 10.22122/arya.v16i3.1938 33447259 PMC7778509

[B27] Mohammadpour R. Yazdimamaghani M. Cheney D. L. Jedrzkiewicz J. Ghandehari H. (2019). Subchronic toxicity of silica nanoparticles as a function of size and porosity. J. Control. Release 304, 216–232. 10.1016/j.jconrel.2019.04.041 31047961 PMC6681828

[B28] Munuera Martinez L. (2010). Total arthroplasty: The other surfaces; wear and tear and osteolysis. An. R. Acad. Nac. Med. 127 (2), 371–380. discussion 381-7. 21877415

[B29] Nguyen T. P. T. Li F. Y. Shrestha S. Tuan R. S. Thissen H. Forsythe J. S. (2021). Cell-laden injectable microgels: Current status and future prospects for cartilage regeneration. Biomaterials 279, 121214. 10.1016/j.biomaterials.2021.121214 34736147

[B30] Saifi M. A. Khan W. Godugu C. (2018). Cytotoxicity of nanomaterials: Using nanotoxicology to address the safety concerns of nanoparticles. Pharm. Nanotechnol. 6 (1), 3–16. 10.2174/2211738505666171023152928 29065848

[B31] Shi D. Xu X. Ye Y. Song K. Cheng Y. Di J. (2016). Photo-cross-Linked scaffold with kartogenin-encapsulated nanoparticles for cartilage regeneration. ACS Nano 10 (1), 1292–1299. 10.1021/acsnano.5b06663 26757419

[B32] Shi W. L. Sun M. Y. Hu X. Q. Ren B. Cheng J. Li C. X. (2017). Structurally and functionally optimized silk-fibroin-gelatin scaffold using 3D printing to repair cartilage injury *in vitro* and *in vivo* . Adv. Mat. 29 (29), 1701089. 10.1002/adma.201701089 28585319

[B33] Shu C. C. Zaki S. Ravi V. Schiavinato A. Smith M. M. Little C. B. (2020). The relationship between synovial inflammation, structural pathology, and pain in post-traumatic osteoarthritis: Differential effect of stem cell and hyaluronan treatment. Arthritis Res. Ther. 22 (1), 29. 10.1186/s13075-020-2117-2 32059749 PMC7023816

[B34] Sun Y. Liu W. Zhang H. Li H. Liu J. Zhang F. (2017). Curcumin prevents osteoarthritis by inhibiting the activation of inflammasome NLRP3. J. Interferon Cytokine Res. 37 (10), 449–455. 10.1089/jir.2017.0069 29028430

[B35] Sundar Dhilip Kumar S. Houreld N. N. Abrahamse H. (2018). Therapeutic potential and recent advances of curcumin in the treatment of aging-associated diseases. Molecules 23 (4), 835. 10.3390/molecules23040835 PMC6017430 29621160

[B36] Tiwari N. Nawale L. Sarkar D. Badiger M. V. (2017). Carboxymethyl cellulose-grafted mesoporous silica hybrid nanogels for enhanced cellular uptake and release of curcumin. Gels 3 (1), 8. 10.3390/gels3010008 PMC6318674 30920505

[B37] Veiseh O. Vegas A. J. (2019). Domesticating the foreign body response: Recent advances and applications. Adv. Drug Deliv. Rev. 144, 148–161. 10.1016/j.addr.2019.08.010 31491445 PMC6774350

[B38] Volpatti L. R. Facklam A. L. Cortinas A. B. Lu Y. C. Matranga M. A. MacIsaac C. (2021). Microgel encapsulated nanoparticles for glucose-responsive insulin delivery. Biomaterials 267, 120458. 10.1016/j.biomaterials.2020.120458 33197650

[B39] Wei W. Ma Y. Z. Zhang X. Z. Zhou W. Y. Wu H. W. Zhang J. W. (2021). Biomimetic joint paint for efficient cartilage repair by simultaneously regulating cartilage degeneration and regeneration in pigs. ACS Appl. Mat. Interfaces 13 (46), 54801–54816. 10.1021/acsami.1c17629 34706537

[B40] Wu B. Li Y. Nie N. Xu J. An C. Liu Y. (2019). Nano genome altas (NGA) of body wide organ responses. Biomaterials 205, 38–49. 10.1016/j.biomaterials.2019.03.019 30903824

[B41] Yang W. Sun C. He S. Q. Chen J. Y. Wang Y. Zhuo Q. (2021). The efficacy and safety of disease-modifying osteoarthritis drugs for knee and hip osteoarthritis-a systematic review and network meta-analysis. J. Gen. Intern Med. 36 (7), 2085–2093. 10.1007/s11606-021-06755-z 33846938 PMC8298729

[B42] Young N. A. Bruss M. Gardner M. Willis W. Mo X. K. Valiente G. (2014). Oral administration of nano-emulsion curcumin in mice suppresses inflammatory-induced NFkB signaling and macrophage migration. Arthritis & Rheumatology 66, S529. 10.1371/journal.pone.0111559 PMC4219720 25369140

[B43] Young N. A. Bruss M. S. Gardner M. Willis W. L. Mo X. Valiente G. R. (2014). Oral administration of nano-emulsion curcumin in mice suppresses inflammatory-induced NFκB signaling and macrophage migration. Plos One 9 (11), e111559. 10.1371/journal.pone.0111559 25369140 PMC4219720

[B44] Zhang C. Cai Y. Z. Lin X. J. (2016). Autologous chondrocyte implantation: Is it likely to become a saviour of large-sized and full-thickness cartilage defect in young adult knee? Knee Surg. Sports Traumatol. Arthrosc. 24 (5), 1643–1650. 10.1007/s00167-015-3643-3 25986097

[B45] Zhang Y. Li S. J. Jin P. S. Shang T. Sun R. Z. Lu L. Y. (2022). Dual functions of microRNA-17 in maintaining cartilage homeostasis and protection against osteoarthritis. Nat. Commun. 13 (1), 2447. 10.1038/s41467-022-30119-8 35508470 PMC9068604

[B46] Zhang Z. Leong D. J. Xu L. He Z. Wang A. Navati M. (2016). Curcumin slows osteoarthritis progression and relieves osteoarthritis-associated pain symptoms in a post-traumatic osteoarthritis mouse model. Arthritis Res. Ther. 18 (1), 128. 10.1186/s13075-016-1025-y 27260322 PMC4891896

[B47] Zheng Z. Sun Y. Liu Z. Zhang M. Li C. Cai H. (2015). The effect of curcumin and its nanoformulation on adjuvant-induced arthritis in rats. Drug Des. devel. Ther. 9, 4931–4942. 10.2147/dddt.s90147 PMC4555965 26345159

